# Development of an *in vitro* periodontal biofilm model for assessing antimicrobial and host modulatory effects of bioactive molecules

**DOI:** 10.1186/1472-6831-14-80

**Published:** 2014-06-28

**Authors:** Emma Millhouse, Anto Jose, Leighann Sherry, David F Lappin, Nisha Patel, Andrew M Middleton, Jonathan Pratten, Shauna Culshaw, Gordon Ramage

**Affiliations:** 1 Infection and Immunity Research Group, Glasgow Dental School, School of Medicine, College of Medical, Veterinary and Life Sciences, University of Glasgow, 378 Sauchiehall Street, Glasgow G2 3JZ, UK; 2 Gum Health and Dry Mouth Group, GlaxoSmithKline Consumer Healthcare, Weybridge, Surrey, UK

**Keywords:** Periodontal disease, Biofilm, Epithelial cell

## Abstract

**Background:**

Inflammation within the oral cavity occurs due to dysregulation between microbial biofilms and the host response. Understanding how different oral hygiene products influence inflammatory properties is important for the development of new products. Therefore, creation of a robust host-pathogen biofilm platform capable of evaluating novel oral healthcare compounds is an attractive option. We therefore devised a multi-species biofilm co-culture model to evaluate the naturally derived polyphenol resveratrol (RSV) and gold standard chlorhexidine (CHX) with respect to anti-biofilm and anti-inflammatory properties.

**Methods:**

An *in vitro* multi-species biofilm containing *S. mitis, F. nucleatum, P. gingivalis* and *A. actinomycetemcomitans* was created to represent a disease-associated biofilm and the oral epithelial cell in OKF6-TERT2. Cytotoxicity studies were performed using RSV and CHX. Multi-species biofilms were either treated with either molecule, or alternatively epithelial cells were treated with these prior to biofilm co-culture. Biofilm composition was evaluated and inflammatory responses quantified at a transcriptional and protein level.

**Results:**

CHX was toxic to epithelial cells and multi-species biofilms at concentrations ranging from 0.01-0.2%. RSV did not effect multi-species biofilm composition, but was toxic to epithelial cells at concentrations greater than 0.01%. In co-culture, CHX-treated biofilms resulted in down regulation of the inflammatory chemokine IL-8 at both mRNA and protein level. RSV-treated epithelial cells in co-culture were down-regulated in the release of IL-8 protein, but not mRNA.

**Conclusions:**

CHX possesses potent bactericidal properties, which may impact downstream inflammatory mediators. RSV does not appear to have bactericidal properties against multi-species biofilms, however it did appear to supress epithelial cells from releasing inflammatory mediators. This study demonstrates the potential to understand the mechanisms by which different oral hygiene products may influence gingival inflammation, thereby validating the use of a biofilm co-culture model.

## Background

Periodontal disease occurs from a dysregulation between the bacterial biofilm at the gum margin and the immune response, resulting in irreversible destruction of both soft and hard tissues supporting the teeth, ultimately leading to tooth loss. In health, innate and adaptive immune molecules mediate the equilibrium between the host and the predominantly Gram positive heterogeneous bacterial suspensions in saliva and adherent biofilm communities on soft and hard tissue surfaces throughout the oral cavity. In periodontal disease the shift in the microbiota from Gram positive to Gram negative species leads to an dysregulated host response both from local tissues and immune cells which induces inflammation and creates a niche at the root for anaerobic species to survive, further exacerbating the disease [1].

Numerous prokaryote species have been identified within the oral cavity existing within complex biofilm ecosystems either as supra- or sub-gingival plaque. Many are uncultivated and unnamed, but all playing important structural and functional roles [2]. Microarray and next generation sequencing studies of the oral microbiota has allowed classification of some species of bacteria into complexes based on associations with health and disease [3,4]. These have allowed studies to investigate species, such as the disease-associated bacteria *Porphyromonas gingivalis*, and understand their role in challenging the host response [5]. However, oral biofilms are complex and within these polymicrobial biofilm structures a myriad of intimate interactions occur, making it difficult to delineate the precise triggers for the pathogenic outcomes associated with immune dysregulation. Therefore, creating a simplified model with several of these key pathogens is an attractive proposition to evaluate host-pathogen interactions and test actives for treatment potential.

Treating periodontal disease is difficult; dentists perform debridement on teeth, administration of antimicrobial mouthwashes to target biofilms and in advanced periodontitis, surgery. However, successes of treatment are variable and on an individual basis and suggesting biofilm composition may influence the outcome of treatment [6]. Antimicrobial mouthwashes currently employed to manage oral diseases include a variety of compounds with chlorhexidine (CHX) being considered the ‘gold standard’ due to its bactericidal and bacteriostatic properties [7]. Additionally, polyphenols, found naturally occurring in plants such as grapes have become a focus for oral therapies due to their anti-inflammatory and anti-bacterial properties [8]. Resveratrol (RSV) is a naturally derived polyphenol, which has been shown to have potent anti-inflammatory properties in a variety of cancer cells and recently periodontitis in rats [9,10].

Both CHX and RSV have desirable properties for periodontitis prevention, being antimicrobial or anti-inflammatory and antimicrobial, respectively. The purpose of this study was therefore to create and validate a multi-species biofilm model to be used in co-culture with host epithelial cells in order to test the actives CHX and RSV in order to validate whether the model system could be used as a sensitive method of delineating their basic modes of action. Here we report that oral epithelial cells produce a reproducible pro-inflammatory response to our multi-species biofilm at both gene and protein level. Additionally the treatment of either epithelial cells or multi-species biofilms with actives resulted in an alteration of inflammation within the co-culture model.

## Methods

### Growth and standardisation of bacteria

*Porphyromonas gingivalis* ATCC 33277*, Fusobacterium nucleatum* ATCC 10953, *Aggregatibacter actinomycetemcomitans* ATCC 43718 and *Streptococcus mitis* ATCC 12261 were used in the course of these studies. *P. gingivalis* ATCC 33277 and *F. nucleatum* ATCC 10596 were grown at 37°C in Schaedler anaerobe broth (Oxoid, Cambridge, UK) for 2 days and 1 day, respectively, in an anaerobic chamber (85% N_2_, 10% CO_2_ and 5% H_2_, [Don Whitley Scientific Limited, Shipley, UK]). *A. actinomycetemcomitans* ATCC 43718 and *Streptococcus mitis* ATCC 12261 were grown at 37°C in tryptic soy broth (Sigma, Poole, UK) supplemented with 0.8% w/v glucose (BDH, Poole, UK) and 0.6% w/v yeast extract (Oxoid, Cambridge, UK) for 1 day in 5% CO_2_. The bacteria were washed with PBS then standardized to an OD_550_ of 0.2, except for *S. mitis*, which was standardized to an OD_550_ of 0.5, in a colorimeter to obtain approximately 1 × 10^8^ cfu/mL of each bacterial species on their specified day of use.

### Active compounds

Chlorhexidine (CHX [Sigma]) and resveratrol (RSV [Sigma]) were used throughout this study. CHX solution was prepared at 0.01, 0.05 and 0.2% v/v in keratinocyte serum free media (KSFM) and used for subsequent antimicrobial testing. RSV powder was solubilised in ddH_2_O prior to preparation in KSFM at 0.01, 0.05 and 0.5% v/v and used for subsequent cell stimulation studies.

### Development of multi-species biofilms

**Figure 1:**
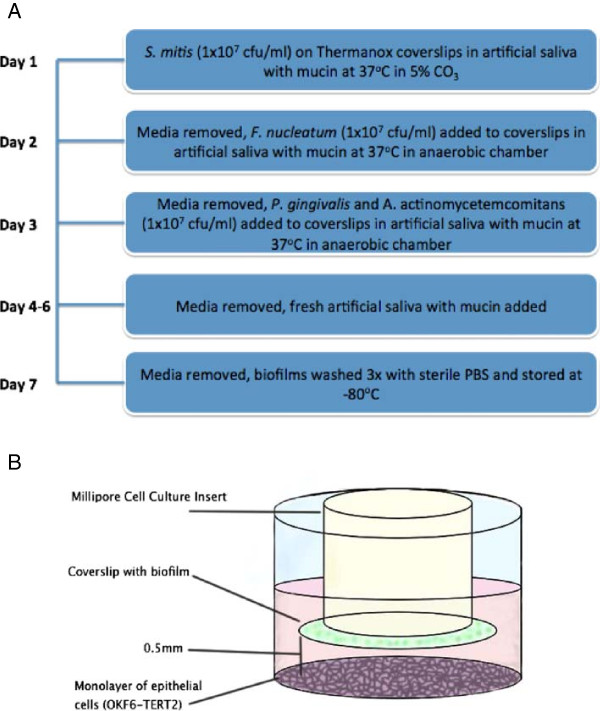
**Development of multi-species biofilm co-culture model. A**. Multi-species biofilm culture protocol. Thermanox™ coverslips were placed in 24 well plates for biofilm culture. Bacteria were grown on appropriate agar plates and cultured in broth for 1–2 days. Cultures were then washed three times in PBS and standardized at 1×10^9^ CFU/ ml, added to artificial saliva to make a 1×10^8^ CFU/ml final volume and added to the biofilm on the appropriate days and cultured in aerobic and anaerobic conditions. After 7 days culture artificial saliva is removed from mature biofilms which are then washed with PBS and stored at -80°C. **B**. Hanging basket co-culture model. Multi-species biofilms were grown on Thermanox™ coverslips and previously described. Epithelial cells (OKF6-TERT2) were seeded in 24 well plates at 1×10^5^ cells/ml in cell culture media. Biofilms were attached inversely to Millipore cell culture inserts using Vaseline® and placed over wells containing cells. Cells and biofilms were co-cultured for 4 and 24 h.

Bacteria were standardized (1 × 10^7^ cfu/mL) in artificial saliva (AS), which contained the following constituents, as described previously [11]. This included porcine stomach mucins (0.25% w/v), sodium chloride (0.35 w/v), potassium chloride (0.02 w/v), calcium chloride dihydrate (0.02 w/v), yeast extract (0.2 w/v), lab lemco powder (0.1 w/v), proteose peptone (0.5 w/v) in ddH_2_O (Sigma, Poole, UK). Urea was then added to independently to a final concentration of 0.05% (v/v). To initiate multispecies biofilm development the pioneer species *S. mitis* biofilm were first formed for 24 h in 5% CO_2_ on 13 mm diameter Thermanox™ coverslips within 24 well plates (Corning, NY, USA). The supernatant was then removed and *F. nucleatum* added, which was incubated anaerobically at 37°C for a further 24 h. The supernatant was removed and *P. gingivalis* and *A. actinomycetemcomitans* added to the dual species biofilm, which was incubated anaerobically at 37°C for a further 4 days, replacing the AS daily to produce a mixed four species biofilm (Figure 1A).

### Quantitative analysis of biofilm composition

Real-time quantitative PCR (qPCR) was then performed to enumerate the definitive and relative composition of the biofilms. Briefly, bacterial biofilms were removed by sonication in a sonic bath at 35 kHz for 10 min, as previously described [12]. For qPCR the biofilm sonicate was used for DNA extraction using the MasterPure Gram Positive DNA Purificiation Kit (Epicentre®, Cambridge, UK), following manufacturers instructions, with the modification that the sonicate was incubated for 4 h to ensure cell lysis. The extracted DNA underwent quality checks using the NanoDrop spectrophotometer (Fischer Scientific, Loughborough). Briefly, 1 μL of extracted DNA was added to a mastermix containing 12.5 μL SYBR® GreenER™, 9.5 μL UV-treated RNase-free water and 1 μL of 10 μM forward/reverse primers for each bacterial species. The primers used were previously published, as listed in Table 1. Three independent replicates from each parameter were analysed in triplicate using MxProP Quantitative PCR machine and MxProP 3000 software (Stratagene, Amsterdam, Netherlands). Samples were quantified to calculate the colony forming equivalent (CFE) based upon a previously established standard curve of bacterial colony forming units ranging from 1 × 10^3^ to 10^8^ cfu/mL. The R^2^ values for these standard curves ranged from 0.956 to 0.994. Melting curve analysis was performed for all primer sets to ensure a single peak, which was indicative of primer specificity. Biofilm architecture was subsequently analysed by scanning electron microscopy (SEM). For this biofilm specimens were washed in PBS, fixed in 2% para-formaldehyde, 2% gluteraldehyde, 0.15% w/v Alcian Blue in 0.15 M sodium cacodylate (pH 7.4) [13]. The fixed and dried samples were sputter-coated with gold and viewed under a JEOL JSM-6400 scanning electron microscope [14].

**Table 1:** Primers used for qPCR in this study

**Gene**	**Forward 5′-3′**	**Reverse 5′-3′**	**Reference**
**Cytokine**			
IL-8	CAGAGACAGCAGAGCACACAA	TTAGCACTCCTTGGCAAAAC	[15]
GAPDH	CAAGGCTGAGAACGGGAAG	GGTGGTGAAGACGCCAGT	[15]
**Bacterial species**			
*S. mitis*	GATACATAGCCGACCTGAG	CCATTGCCGAAGATTCC	[16]
*F. nucleatum*	GGATTTATTGGGCGTAAAGC	GGCATTCCTACAAATATCTACGAA	[17]
*P. gingivalis*	GCGCTCAACGTTCAGCC	CACGAATTCGCCTGC	[18]
*A. actinomycetemcomitans*	GAACCTTACCTACTCTTGACATCCGAA	TGCAGCACCTGTCTCAAAGC	[19]

### Development of an epithelial biofilm co-culture model

OKF6-TERT2 cells (kind gift of the Rheinwald laboratory, Brigham and Woman’s Hospital, Boston) are an immortalized human oral keratinocyte cell line [20] was used throughout these investigations. OKF6 cells were cultured in keratinocyte serum-free medium (KSFM) as previously described [21]. At 90% confluence the cells were trypsinised, washed in Hanks balanced salt solution then re-seeded to approximately 1 × 10^5^ cells/mL and seeded onto a Thermanox™ coverslip within a 24 well cell culture plate (Corning, NY, USA). The epithelial cells were washed and then subject to challenge with inverted biofilms, attached using Vaseline® to hanging cell culture inserts (Millipore, MA, USA) as illustrated in Figure 1B. The biofilms on the Thermanox™ discs were separated from the epithelial cells on the bottom of the well by a small space of <0.5 mm, representative of a gingival crevice. Multispecies biofilms were incubated in the co-culture model for 4 and 24 h and the viability of the epithelial cells ± active treatments (CHX and RSV) evaluated using a metabolic assay of 10% v/v alamarBlue®, according to the manufacturer’s instructions (Life Technologies, Paisley, UK). After 4 h incubation the absorbance was read at 570 nm and the reference wavelength at 600 nm, and the percentage reduction in biofilm viability calculated using the manufacturer’s formula.

### Assessing inflammatory changes during biofilm co-culture

Supernatants and cell lysates were collected from OKF6/TERT2 cells stimulated with different multispecies biofilms, which were used for protein and transcriptional analysis, respectively, to assess the regulation of pro-inflammatory mediators. Initial gene expression analysis was carried out using a custom designed RT^2^ Profiler PCR Array (Qiagen, Crawley, UK). RT^2^ Profiler arrays are a SYBR® GreenER™ based real-time PCR that allow for the detection of several genes of interest, simultaneously. Briefly, RNA was extracted from cell lysates (Qiagen, Crawley, UK) and 55 ng/μl of cDNA synthesised using the RT^2^ First Strand cDNA synthesis kit (Qiagen, Crawley, UK), as per manufacturers instructions. Briefly, 24 μl of a mastermix containing SYBR® GreenER™, cDNA synthesised using the RT^2^ First Strand kit (Qiagen) and RNase-free water was added to each well of the RT^2^ Profiler plate, which already contained the forward and reverse primers for the genes of interest (IL-1α, IL-1β, IL-6, TNF, CSF2, CSF3, IL-8, CXCL1, CXCL3, CXCL5, CCL1 and GAPDH), based on an experimental gingivitis model [22]. Two replicates of each condition were used in the RT^2^ Profiler, which was carried out on two separate occasions.

Further verification was performed using IL-8 gene expression analysed using SYBR® Green based qPCR (Invitrogen), using GAPDH as a housekeeping gene. Primer sequences and reference sources are listed in Table 1. All primers were tested against each bacterial species to ensure specificity, which was the case (data not shown). RNA was synthesised into cDNA then added to a mastermix containing 12.5 μl SYBR® GreenER™, 10.5 μl UV-treated RNase-free water and 0.5 μl of forward/reverse primers. Three independent replicates from each parameter were analysed in duplicate using MxProP Quantitative PCR machine and MxProP 3000 software (Stratagene, Amsterdam, Netherlands) and gene expression normalised to the housekeeping gene GAPDH according to the 2^*-ΔΔCT*^ method [23]. IL-8 release into cell culture supernatants was assessed by ELISA (Invitrogen, Paisley, UK), as per manufacturer’s instructions. Results were calculated using a 4-parameter curve fit, quantifying colometric changes at 630 nm (BMG-Labtech, Ortenberg, Germany).

### Statistical analysis

Graph production, data distribution and statistical analysis were performed using GraphPad Prism (version 4; La Jolla, CA, USA). After assessing whether data conformed to a normal distribution by before and after data transforms, One-way Analysis of Variance (ANOVA) and *t* tests were used to investigate significant differences between independent groups of data that approximated to a Gaussian distribution. A Bonferroni correction was applied to the p value to account for multiple comparisons of the data. Non-parametric data was analysed using the Mann–Whitney U-test to assess differences between two independent sample groups. Student t-tests were used to measure statistical differences between the *Δ*Ct values of the two independent groups assessed in gene expression studies, although data may be represented as percentage or fold change in the figures. Statistical significance was achieved if *P < 0.05*.

## Results

### Quantitative analysis of a multi-species periodontal biofilm model

**Figure 2:**
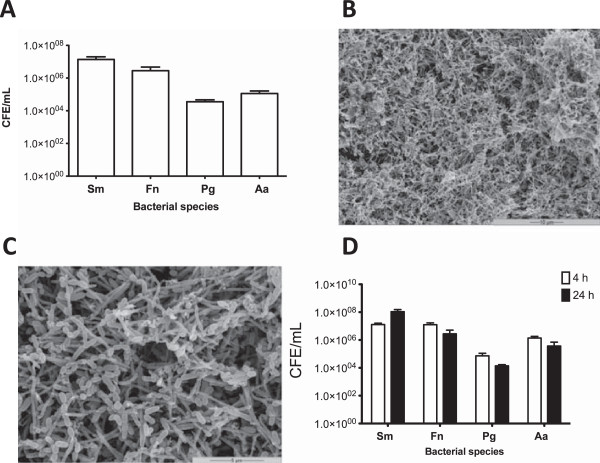
**Analysis of multi-species biofilms in anaerobic and aerobic conditions.** Multi-species biofilm were grown on Thermanox™ coverslips in 24 well plates for 6 days as previously described. After maturation DNA was extracted using Masterpure™ Gram-positive DNA purification kit for quantification of each species using SYBR® GreenER™ based qPCR **(A)**. Biofilm morphology was analyzed by SEM at 2000x **(B)** and 5000x **(C)**. Samples were processed and viewed on a JEOL JSM-6400 scanning electron microscope and images assembled using Photoshop software. Biofilms can be seen to be complex **(B)**. At higher magnifications different morphologies can be identified with *S. mitis* and *F. nucleatum* making up the majority of the biofilm **(C)**. Mature biofilms were also cultured in cell culture media in 5% CO_2_ for 4 and 24 h before DNA extraction and quantification of each species using using SYBR® GreenER™ based qPCR **(D)**. All samples were assayed in triplicate on three separate occasions. Data are mean ± SD.

Sonicated multi-species biofilms were quantified by qPCR (Figure 2A). It was shown that quantitatively that *S. mitis* was the most dominant species within the mature biofilm (1.36 × 10^7^ CFE/mL; 83.24%)*,* followed by *F. nucleatum* (2.28 × 10^6^; 15.16%)*, P. gingivalis* (3.53 ×10 ^4^; 1.01%) and *A. actinomycetemcomitans* (1.13 × 10^5^; 0.58%). The biofilm composition was then examined using SEM analysis. The biofilm was shown to be a dense complex of different morphotypes dominated by *F. nucleatum* (Figure 2B and C).

In order to test the multi-species biofilms in co-culture with epithelial cells, we examined the impact of moving the biofilm from anaerobic conditions within AS to 5% CO_2_ in KSFM. To do this we evaluated the biofilm composition as described above. No significant differences were observed in the composition and quantity of the 4 species within the CO_2_ biofilm at 4 and 24 h (Figure 2D). There was also no significant difference between 24 h biofilms formed under anaerobic conditions and those then placed in CO_2_ for a further 24 h. These biofilms were then used in a co-culture system to investigate the biological properties of two different bioactive agents.

### Investigating the effects of bioactive agents in a multi-species biofilm epithelial cell co-culture model

**Figure 3:**
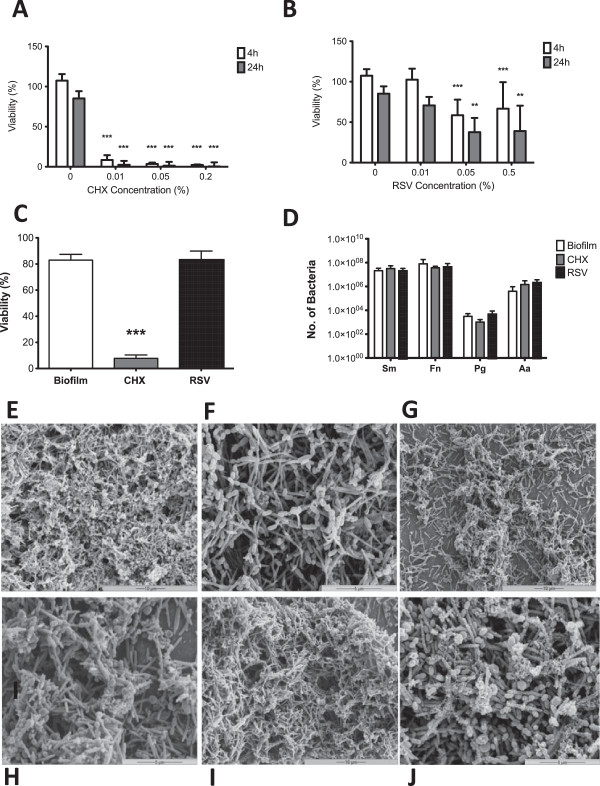
**Direct effect of actives on oral epithelial cells and multi-species biofilms.** The oral epithelial cell line OKF6-TERT2 was seeded at 1×10^5^ cells/ ml in 24 well plates for toxicity studies. Cells were treated with concentrations of CHX (0.01, 0.05, 0.2% v/v) **(A)** and RSV (0.01, 0.05, 0.5% w/v) **(B)** for 30 minutes before washing with PBS. Cell viability was assessed using the Alamarblue® assay with absorbance read at 570 nm and 600 nm. Following this concentrations chosen for the remainder of the study were CHX 0.2% (v/v) and RSV 0.01% (w/v). To investigate the role of actives in biofilm treatment biofilms were grown on coverslips in 24 well plates for 6 days as previously described. Mature biofilms were treated with 0.2% v/v CHX and 0.01% w/v RSV for 30 minutes before washing with PBS. Biofilm viability was measured using Alamarblue® read at 570 nm and 600 nm **(C)**. Bacterial DNA was extracted and each species quantified using SYBR® GreenER™ based qPCR **(D)**. Biofilms were also analyzed by SEM at both 2000x **(E, G, I)** and 5000× **(F, H, J)**. Samples were processed and viewed on a JEOL JSM-6400 scanning electron microscope and images assembled using Photoshop software. Untreated biofilms **(E, F)** were compared with biofilms treated with 0.2% (v/v) CHX **(G, H)** and and 0.01% (w/v) RSV **(I, J)**. Samples were assayed in triplicate on three separate occasions and data are mean ± SD (**p < 0.01, ***p < 0.001).

First, to optimise the concentrations of RSV and CHX to be tested in this model we undertook cytoxicity tests on both epithelial cells and on biofilms.Oral epithelial cells were treated for 30 min with three concentration of the antimicrobial active CHX (0.01, 0.05, 0.2% v/v) and anti-inflammatory active RSV (0.01, 0.05, 0.5% w/v) before washing with PBS and incubated for 4 and 24 h before cell viability was measured assessed using an AlamarBlue® assay. The data showed a significant decrease (p < 0.001) in cell viability compared to the untreated media controls when epithelial cells are incubated with CHX at any concentration for both 4 and 24 h (Figure 3A). When epithelial cells were co-cultured with RSV significant decreases in cell viability compared with the media control were observed using 0.05 and 0.5% w/v RSV concentrations at both 4 (p < 0.001) and 24 h (p < 0.01), with an approximately 50% decrease in cell viability at each time (Figure 3B).Next, from these data the concentrations taken forward for the remainder of the study were 0.2% v/v CHX and 0.01% w/v RSV. Multi-species biofilms were treated with the chosen concentrations of actives for 30 min and biofilm viability measured using an AlamarBlue® assay (Figure 3C). Treatment of the multi-species biofilm with RSV did not significantly affect biofilm viability compared to the untreated biofilm control. However, treatment with CHX showed a significant reduction (p < 0.001) of 75% in bacteria viability compared with the untreated biofilm.To determine if treatment with these actives altered the species composition of the biofilm, they were treated for 30 min then DNA extracted and quantification of each species performed by qPCR (Figure 3D). The data show no significant change in the composition following a 30 min treatment with CHX or RSV compared with the untreated biofilm. To further investigate this effect SEM analysis was performed to examine the impact of each active on the architecture of the biofilms post-treatment (Figure 3E-J). CHX appeared to destabilise the biofilm, as the complexity of the biofilm was reduced, whereas RSV appeared to have no visual effect on architecture.

**Figure 4:**
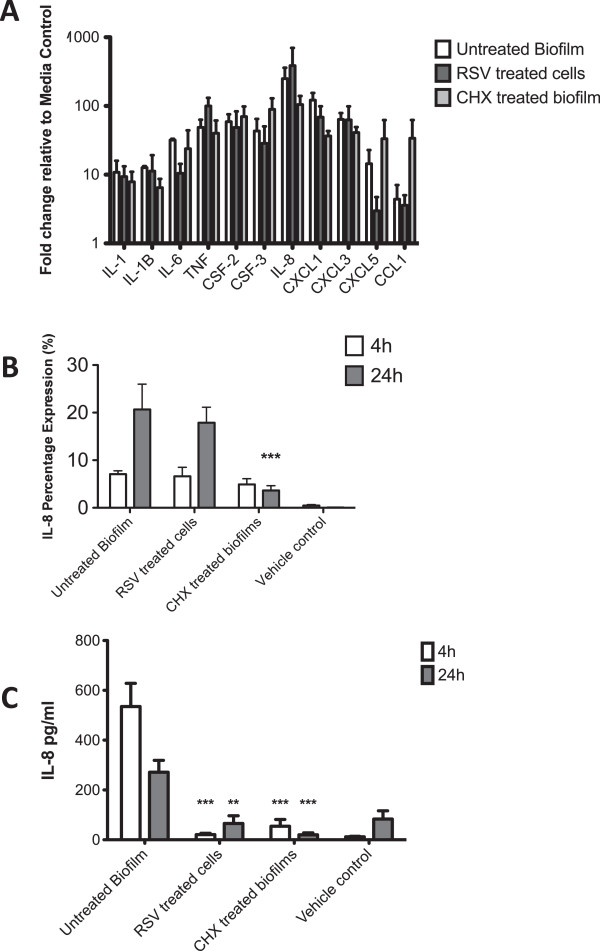
**CHX and RSV immunomodulate epithelial cell responses to multi-species biofilm co-culture *in vitro.*** The oral epithelial cell line OKF6-TERT2 was seeded at 1×10^5^ cells/ml in 24 well plates for co-culture with multi-species biofilms. Biofilm were pre treated with 0.2% (v/v) CHX or cells were treated with 0.01% (w/v) RSV for 30 minutes before washing with PBS and then co-cultured with untreated biofilms or cells respectively for 4 and 24 h. Untreated control co-culture were also included. RNA was extracted from the cell lysates at 4 h, cDNA was synthesized and pro-inflammatory gene expression quantified on an RT^2^ profiler plate **(A)**. Duplicate samples from three independent experiments were used and data are mean ± SD relative to media control. Samples were also assayed for IL-8 relative to housekeeping gene GAPDH at 4 and 24 h using using SYBR® GreenER™ based qPCR **(B)**. Supernatants were also analyzed for exogenous IL-8, measured by ELISA **(C)**. Samples were assayed in triplicate from three independent experiments, data are mean ± SD (**p < 0.01, ***p < 0.001).

Next, we evaluated the effects of the untreated multi-species biofilm stimulated OKF6 cells to ensure the biofilm induced inflammatory mediators. Using the RT^2^ Profiler we compared biofilm stimulated cells to media control cells after 4 h to determine the inflammatory characteristics of the model (Table 2). Significant increases were observed for all the genes upon the RT^2^ profiler selected based on their expression during induced experimental gingivitis in human subjects, ranging from 4.39 fold change (CCL1) to 249.8 fold change (IL-8), with an mean fold change of 60 compared to the unstimulated media control. These data were verified by investigating IL-8 using specific primers within a reverse transcriptase (RT) qPCR assay (Figure 4B). A significant increased was observed of a 15.57 fold change (p < 0.001) after 4 h and 312.88 fold change (p < 0.001) after 24 h when compared to the media control (Table 2). Finally, we evaluated the cell supernatants for the release of IL-8 within this system, where it was shown that after 4 h (535 ng) and 24 h (450 ng) that IL-8 release was significantly increased (p < 0.001) compared to the untreated media controls (Figure 4C). Based on this collective data, we have demonstrated that the 4-species biofilm has reproducible inflammatory properties within an epithelial co-culture model.

**Table 2:** Pro-inflammatory response to untreated biofilms

**Gene**	**Average fold increase**	**SD (±)**	**Significance**
IL-1	10.820	8.918	p < 0.05
IL-1B	12.662	1.161	p < 0.05
IL-6	31.876	2.366	p < 0.001
TNF	48.821	24.623	p < 0.001
CSF-2	59.200	28.294	p < 0.001
CSF-3	43.331	37.126	P < 0.001
IL-8	249.805	189.93	p < 0.001
CXCL1	121.892	56.676	p < 0.001
CXCL3	63.843	26.213	p < 0.001
CXCL5	14.435	14.474	n/s
CCL1	4.397	4.629	n/s

We then determined how CHX treatment of the biofilm affected inflammation. The co-culture model was utilised with biofilms treated with 0.2% CHX for 30 min. Following co-culture epithelial cell lysates and supernatants were removed to be tested for increased gene and protein inflammatory markers. An untreated biofilm and epithelial cells not co-cultured with the multi-species biofilm were used as controls. After 4 h co-stimulation with the CHX treated biofilms we showed no significant difference in gene expression using the RT^2^ profiler, though IL-8 and CXCL1 were notably decreased (Figure 4A). Further analysis of IL-8 using qPCR again showed no significant difference in expression at 4 h, whereas at 24 h a significant decrease of 5.65 fold was observed in the CHX treated biofilm stimulated cells (p > 0.001) (Figure 4B). IL-8 protein expression was also measured at 4 and 24 h by ELISA. A significant reduction (p < 0.001) of IL-8 protein was observed at 4 h (10 fold) and at 24 h (13 fold) when compared to the untreated control (Figure 4C).

Finally, we evaluated the effect of RSV treated epithelial cells in the co-culture model. Epithelial cells were treated for 30 min with RSV, washed and co-incubated with untreated biofilms for 4 and 24 h. The RT^2^ profiler showed no significant differences in gene expression after 4 h compared with untreated biofilms (Figure 4A). Further analysis of IL-8 at 4 and 24 h also showed no significant differences in expression levels (Figure 4B). However, a significant decrease in IL-8 protein levels at 4 h (25 fold; p < 0.001) and 24 h (13 fold; p < 0.01) were observed when compared to the untreated biofilm co-culture model (Figure 4C).

## Discussion

Using experimental *in vitro* biofilm models to investigate interactions between biofilms and host epithelial cells is essential to understanding the mechanisms of disease pathology and how actives can influence the host response. Using this bespoke multi-species biofilm model we have demonstrated that the epithelial inflammatory response to multi-species biofilms can be altered in the presence of antimicrobial and anti-inflammatory compounds.

The data show co-culture of epithelial cells and untreated multi-species biofilms produce a significant increase in both gene and protein expression at 4 and 24 h. IL-8 protein levels were significantly increased at both 4 and 24 h in co-culture of epithelial cells and untreated multi-species biofilms compared with the cells only control. Additionally, increases in gene expression of a variety of pro-inflammatory chemokines and cytokines including IL-6, IL-8, TNF, CSF-2, CXCL1 and CXCL3 at 4 h compared with the cells only control were observed. These dynamic changes in pro-inflammatory mediators demonstrate that there is interplay between the complex biofilm and the epithelial cells. Notably, at 24 h the RNA quality from the co-culture model was poor and therefore not used to test gene expression using the RT^2^ profiler assay, however IL-8 was able to be measured at 24 h using SYBR® GreenER™ based qPCR, and was significantly increased compared to media controls. Pro-inflammatory responses by epithelial cells after challenge with oral biofilms have been previously reported using a variety of oral associated bacteria [24,25]. Recently, it has been shown that biofilms including the ‘red complex’ organisms such as *P. gingivalis* or *F. nucleatum* increased IL-8 production during early host interaction, yet with longer exposure the chemotactic factors are downregulated, presumably through the release of proteolytic gingipains [25]. These studies also highlight the essential role of studying epithelial responses to bacteria in a biofilm model compared with planktonic bacteria and suggest biofilms behave differently to their single species counterparts, which may be in part due to the complex biofilm interaction that occur in a microbial community [26].

We have also demonstrated how antimicrobial and anti-inflammatory actives can influence the host response in this co-culture model. CHX while a potent antimicrobial, also has a high toxicity level when used to treat epithelial cells and in this study it was therefore decided to treat the biofilms with CHX before co-culture. Our data show that biofilms treated for 30 min with CHX have significantly decreased bacterial viability. However, differences were observed between the quantitative analysis of the biofilm composition by qPCR and SEM after 30 min treatment with CHX. These are most likely due to the substantivity of CHX where straight after treatment bacteria are dead but not dissociated from the biofilm, which is shown in the qPCR composition. After treatment and 4 h co-culture images by SEM show the biofilm is less complex which may be due to the loss of the dead bacteria from the biofilm as the effects of CHX decrease over time. This data agrees with previous work of the mechanisms of action of CHX which show an immediate bacteriocidal action combined with a prolonged bacteriostatic action due to absorption of the active to the surface [27]. Epithelial cells co-cultured with CHX treated multi-species biofilms did not show a significant change in overall gene expression at 4 h but a significant decrease in IL-8 levels was observed at 24 h. However, many of the other pro-inflammatory molecules did tend to decrease at 4 h, such as IL-6 and IL-1β, though increased others such as CCL1. Levels of IL-8 protein were significantly decreased at both 4 and 24 h. Clinical data of studies using CHX mouthwashes show significant reductions in inflammation following CHX treatment than control groups [28]. This suggests that reduction in inflammatory markers may be time dependent and linked to the disaggregation of dead bacteria from the surface of the biofilm, though the release of bacterial products may inadvertently activate other pathways.

Testing the anti-inflammatory active RSV we observed no significant decrease in biofilm viability or epithelial cell viability at 0.01%. However a significant decrease in epithelial cell viability was observed at concentrations of 0.05% and 0.5%. RSV has been reported to have antimicrobial effects both against planktonic and bacterial biofilms, with most studies focusing on bacteria such as *P. aeruginosa* and *E. coli* with MIC ranging from 5-50 μg/ml [29,30]. There are no previous studies investigating the toxicity of RSV on epithelial cells. However. studies have shown RSV induces apoptosis in cancer cell lines at 0.1-0.3% [31]. Epithelial cells treated with RSV and co-cultured with multi-species biofilms showed no significant differences in overall gene expression compared with the untreated controls, though CXCL5, the neutrophil activating peptide was decreased transcriptionally, which may be involved in reducing neutrophil activation [32]. Moreover, a significant decrease in IL-8 protein expression was observed at 4 and 24 h compared with the untreated control. This suggests that RSV may disrupt the signalling pathway and result in reduced chemokine and cytokine production. A variety of studies investigating the role of RSV and inflammation have found RSV modulates a variety of cell responses by interfering with signalling pathways [33,34]. With regards to our own work previous studies have reported RSV modulates pro-inflammatory responses by modulating NF-κB and AP-1 [35,36].

## Conclusions

This study has investigated the inflammatory response by epithelial cells to oral biofilms in co-culture and how actives can mediate this response. We have shown that multi-species biofilm treatment with the antimicrobial CHX is able to reduce inflammation produced by epithelial cells at both a gene and protein level. Treatment of epithelial cells with the anti-inflammatory compound RSV results in a reduction of epithelial cell inflammation at protein level. Although the main limitation of this model may be the number of species within the biofilm consortium, this does allow more control and reproducibility in the model. However, increasing the complexity of this model is subject to further work by our group. Collectively, this work supports the generation of a validated multi-species periodontal biofilm model suitable for use in the screening and testing of potential oral hygiene actives.

## Competing interests

EM, LS, DFL, SC and GR have no competing interests. AJ, NP, AMM and JP are all employees of GlaxoSmithKline, the sponsor of the study.

## Authors’ contributions

EM, AJ and LS participated in the study design, carried out the experimental studies on biofilms, performed statistical analysis and were responsible for the manuscript. DFL participated in study design, assisted with statistical support and helped draft the manuscript. NP, AMM, JP and SC contributed to study design and supervised manuscript writing. GR conceived the study, participated in study design, data analysis and was responsible for writing and submission of the final manuscript. All authors read and approved the manuscript.

## Authors’ information

Infection and Immunity Research Group, Glasgow Dental School, School of Medicine, College of Medical, Veterinary and Life Sciences, University of Glasgow.

## Pre-publication history

The pre-publication history for this paper can be accessed here:

http://www.biomedcentral.com/1472-6831/14/80/prepub
